# The effect of e-healthy diet literacy on sustainable nutrition behaviours in pregnant women

**DOI:** 10.1017/S1368980026102870

**Published:** 2026-06-16

**Authors:** Şeyma Demir, Yasemin Erkal Aksoy

**Affiliations:** Midwifery, Selcuk University, Türkiye

**Keywords:** Diet, e-healthy, Literacy, Nutrition, Pregnancy

## Abstract

**Objective:**

The aim of this study was to examine the relationship between e-healthy diet literacy and sustainable nutrition behaviours in pregnant women.

**Design:**

This study was a descriptive correlational study.

**Setting:**

Data were collected via self-report using a Personal Information Form, the e-Healthy Diet Literacy Questionnaire (e-HDLQ) and the Sustainable Nutrition Behavior Scale (SNBS).

**Participants:**

The study population consisted of all pregnant women living in Konya/Türkiye, and the sample included 290 pregnant women over the age of 18 years who voluntarily agreed to participate and were able to read and understand Turkish, the language of the questionnaire.

**Results:**

The total e-HDLQ score of the participants was 29·37 (sd 6·91) (min = 13, max = 46), and the total SNBS score was 109·18 (sd 17·07) (min = 37, max = 145). Weak but statistically significant associations were observed between the total e-HDLQ score and the SNBS sub-dimensions of food preference (*r* = 0·119) and food purchase (*r* = 0·117) (*P* < 0·05). Additionally, weak but statistically significant associations were identified between the e-HDLQ appraising sub-dimension and the total SNBS score (*r* = 0·125), its food preference sub-dimension (*r* = 0·134), and food waste reduction sub-dimension (*r* = 0·119) (*P* < 0·05). Multiple linear regression analyses showed that age, education level, history of having a low-birth-weight baby and food preference and food purchase sub-dimensions positively predicted e-HDLQ, whereas knowledge of nutrition and seasonal and local food consumption sub-dimension were negatively associated. For SNBS, the appraising sub-dimension positively predicted behaviour, while higher education level was negatively associated.

**Conclusions:**

The study shows that pregnant women’s e-healthy diet literacy is weakly but significantly associated with various aspects of sustainable nutrition behaviours.

## Graphical abstract

**Figure:**
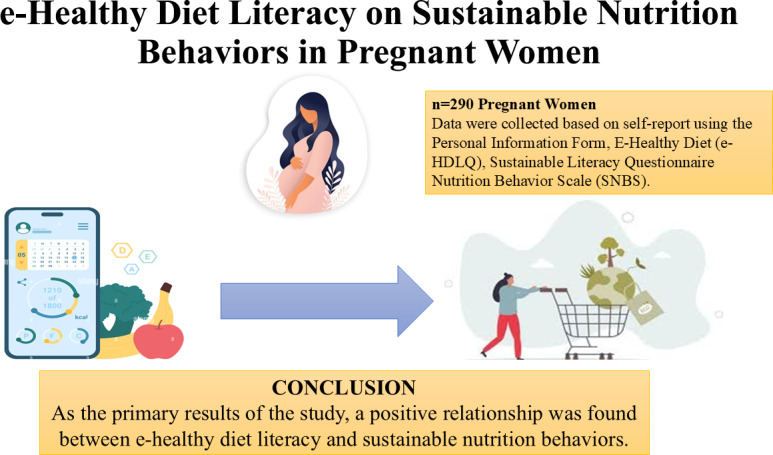

Pregnant women increasingly turn to the internet to access reliable information about healthy eating^(1,2)^. Social media, pregnancy-related mobile applications and other accessible digital resources have become primary sources of information for decision-making and lifestyle changes during pregnancy^(3,4)^. However, not all digital nutrition information is accurate or trustworthy. Therefore, pregnant women need the ability to search, comprehend, appraise and apply online nutrition information correctly, which enables them to make informed dietary decisions^(5)^. In this context, the WHO recommends providing support, counselling and information services to pregnant women^(6)^.

Recent studies indicate that e-healthy diet literacy may influence pregnant women’s dietary behaviours^(7–9)^. However, research on the relationship between e-healthy diet literacy and sustainable nutrition behaviours in pregnant women is limited. Sustainable nutrition during pregnancy is a multidimensional concept, addressing not only the current health needs of the mother and fetus but also the environmental sustainability for future generations^(10)^.

According to the FAO, sustainable nutrition involves using resources efficiently to meet the needs of the present generation without compromising the ability of future generations to meet their own needs^(11)^. Pregnant women, motivated by the instinct to protect their babies’ health, may be more likely to adopt sustainable nutrition principles^(12)^. Food choices, consumption patterns, waste management and environmental impacts during pregnancy may have long-term consequences^(7–9,12)^. Understanding sustainable nutrition behaviours in pregnant women may therefore support maternal and fetal health while promoting environmentally conscious dietary habits.

Nutrition literacy is defined as ‘the degree to which individuals may acquire, process and understand the basic nutritional information needed to make appropriate nutritional decisions’^(13)^. Individuals with high nutrition literacy develop healthy habits, access accurate information, value education and make informed food choices^(14)^. E-healthy diet literacy is defined as the ability to access, understand, evaluate and apply nutrition information obtained through digital platforms. This may be positively associated with sustainable nutritional behaviours during pregnancy^(15)^.

This study was guided by the Integrated Model of Health Literacy proposed by Sørensen *et al.* (2012), which conceptualises health literacy as a dynamic process encompassing individuals’ abilities to access, understand, appraise and apply health-related information to make informed decisions and maintain health behaviours^(16)^. In this context, e-healthy diet literacy represents a digital health competency that enables pregnant women to manage online dietary information effectively, shaping their sustainable nutritional behaviours. Incorporating this theoretical framework provides a clearer understanding of how digital literacy competencies contribute to sustainable health practices during pregnancy. The aim of this study was to evaluate the relationship between e-healthy diet literacy and sustainable nutrition behaviours in pregnant women, thereby filling a gap in the literature and providing a basis for interventions that promote sustainable dietary habits during pregnancy.

## Methods

### Research questions

1. Do the sociodemographic characteristics of pregnant women affect their e-healthy diet literacy?
2. Are there differences in e-healthy diet literacy and sustainable nutrition behaviour scores according to the sociodemographic characteristics of pregnant women?
3. Is there a relationship between e-healthy diet literacy level and sustainable nutrition behaviours of pregnant women?

### Design and participants

This study was a descriptive correlational study. The population consisted of all pregnant women living in Konya/Türkiye. The sample size was calculated using the G * Power 3.1.9.4 program. Based on the known mean score of the e-Healthy Diet Literacy Questionnaire (e-HDLQ) (31·50 (sd 6·6)) reported by Karahan Yılmaz *et al.* (2023), the required sample size was determined as 269 participants with 85 % power and an effect size of 0·15 within a one-point deviation^(17)^. The effect size (f^2^ = 0·15) was chosen according to Cohen’s (1988) criteria for a medium effect size in social science research^(18)^. Considering potential missing data, 295 women were targeted. The study used a convenience sampling method and collected data from pregnant women who met the inclusion criteria and agreed to participate voluntarily during the data collection period. Participants had to meet the following inclusion criteria: be 18 years of age or older; be Turkish speakers capable of reading and understanding Turkish; be smartphone users; be pregnant; and provide informed consent and fully complete the survey questions. Those diagnosed with a high-risk pregnancy, undergoing psychiatric treatment (self-reported) or wishing to withdraw from the study were excluded. The dependent variable of the study was sustainable nutrition behaviours, while the independent variables included e-healthy diet literacy levels, as well as sociodemographic and obstetric characteristics.

### Data collection tools

In the study, data were collected based on self-report using the Personal Information Form, e-HDLQ and Sustainable Nutrition Behavior Scale (SNBS).

#### Personal Information Form

The form prepared by the researcher by reviewing the literature^(19–21)^ consists of a total of nineteen questions including the sociodemographic and obstetric characteristics of the participants and their perceptions and status on nutrition. Sociodemographic characteristics included questions about age, marital status, education level of self and spouse, employment status, social security, family type, place of residence and income status. Obstetric questions included gestational week, number of pregnancies, time elapsed between the last two pregnancies and the birth weight of the baby in the previous delivery. Regarding nutrition, there are questions about whether she received information or education about nutrition during her pregnancy, whether she had heard of the concept of sustainable nutrition before and where she researched the most about nutrition.

#### e-HDLQ

The e-HDLQ was developed by Duong and colleagues in 2020 to measure digital literacy^(22)^. The scale (2023) was adapted into Turkish by Yılmaz *et al.* The e-HDLQ has four sub-dimensions: ‘Accessing’, ‘Understanding’, ‘Appraising’ and ‘Applying’. The Accessing (items 1, 2 and 3) sub-dimension was measured using a five-point Likert-type scale ranging from 1 (never) to 5 (daily); the Understanding (items 4, 5, 6 and 7) sub-dimension was evaluated as follows through yes/no/don’t know options. ‘Yes’ option is worth 5 points, while ‘no’ or ‘don’t know’ statements are worth 1 point. Appraising (items 8 and 9) was measured using a five-point Likert-type scale ranging from 1 (strongly disagree) to 5 (strongly agree). Applying (items 10 and 11) was assessed using a five-point Likert-type scale ranging from 1 (never) to 5 (always). The total score obtained from the scale is calculated by summing the responses to all items, and the scale consists of eleven items. Cronbach’s *α* value was calculated as 0·55^(17)^. Since the scale had already been validated in a Turkish population, no additional cultural or linguistic adaptation was performed for pregnant women in this study. However, the reliability of the scale was reassessed within this study’s sample of pregnant women. The Cronbach’s *α* coefficient for this group was found to be 0·58, which indicates an acceptable level of internal consistency for research purposes.

#### Sustainable Nutrition Behavior Scale

The scale was adapted into Turkish by Garipoglu *et al.* (2023)^(23)^. The Turkish version of the SNBS is a five-point Likert-type scale consisting of twenty-nine items and four sub-dimensions. Items 1, 2, 3, 4, 5 and 6 of the scale are food preference. Items 7, 8, 9, 10, 11, 12, 13, 14 and 15 are on food waste reduction. Items 16, 17, 18, 19, 20, 20, 21, 22 and 23 are on seasonal and local food consumption, and items 24, 25, 26, 27, 28 and 29 are on food purchase. The responses to each option in the items are (1) never, (2) rarely, (3) sometimes, (4) frequently and (5) always and are scored from 1 to 5. The lowest score that can be obtained from the scale is 29 and the highest score is 145, and there are no reverse items in the scale. A higher total score and sub-dimension scores indicate that the individual has more sustainable nutrition behaviours. In this study and in Garipoğlu’s study, the Cronbach’s *α* reliability coefficient of the scale was determined as 0·92^(23)^.

### Statistical analysis

The statistical analysis of the data obtained within the scope of this research was carried out using the SPSS 25.0 package program. Numerical variables were expressed as mean (X) and standard deviation, and qualitative variables were expressed as number (*n*) and percentage (%). Although the e-HDLQ and SNBS are based on Likert-type items, the total scale scores were treated as continuous variables for the purposes of statistical analysis, as this approach is widely accepted in health and social science research when scale scores are summed to produce a total score^(24)^. Skewness and kurtosis tests were used to check whether the variables fit the normal distribution. The skewness and kurtosis values were found to be between +1 and –1. Parametric tests were used to compare quantitative data. Independent groups *t* test was used to compare two-group variables, and ANOVA was used for three-group variables with Tukey’s *post hoc* test. The relationship between the scales was analysed by Pearson’s correlation test. In addition, multiple linear regression analyses were performed using the Backward method to examine the predictors of the e-HDLQ and the SNBS. Two separate models were constructed, with the e-HDLQ and SNBS total scores as the dependent variables, and sociodemographic factors and sub-dimension scores as the independent variables. The assumptions of linearity, independence, homoscedasticity and multicollinearity were checked, and significant predictors were identified. All statistical calculations were interpreted at *P* < 0·05 significance level.

### Data collection

The data of the study were collected in the Konya district of Türkiye. The places where the pregnant women resided were determined according to the information of the district governorship, and face-to-face visits were made. Data were collected based on self-report using Personal Information Form, e-HDLQ and SNBS. It takes an average of 5–10 min to fill out the scale. The form was given to the pregnant women after explaining the purpose of the study. In order to fill out the questionnaire during the home visit, care was taken to create a quiet environment as much as possible, and the questionnaire forms were filled out in a quiet room to minimise distractions (television, radio, children’s voices, etc.). The researcher helped the woman when she had any need.

## Results

A total of 295 pregnant women were invited to participate in the study. Five participants withdrew during the data collection process, leaving a final sample of 290 pregnant women. The mean age of the participants was 27·91 (sd 5·27) (min = 18, max = 46). The total score of e-HDLQ was calculated as 29·37 (sd 6·91) (min = 13 and max = 46), and the total score of SNBS was calculated as 109·18 (sd 17·07) (min = 37, max = 145). In the study, the food purchase sub-dimension score of the participants aged 33 years and over was statistically higher than the participants aged 18–32 years (*P* = 0·003). Among the participants, 26·2 % had completed primary education, 39·0 % had completed secondary education and 34·8 % were graduates of higher education. It was found that there was a significant difference between the educational status of the participants according to their total e-HDLQ (F = 11·354; *P* ˂ 0·001), accessing sub-dimension (F = 14·600; *P* ˂ 0·001), appraising sub-dimension (F = 5·236; *P* = 0·006) and applying sub-dimension (F = 9·269; *P* ˂ 0·001) scores. It was found that 23·8 % of the participants’ spouses were primary school graduates, 40·3 % were secondary school graduates and 35·9 % were higher education graduates. It was found that there was a significant difference between the participants’ spouses education status and total e-HDLQ (F = 7·692; *P* ˂ 0·001), accessing sub-dimension (F = 7·768; *P* ˂ 0·001), appraising sub-dimension (F = 5·302; *P* = 0·005) and applying sub-dimension (F = 8·272; *P* ˂ 0·001) scores.

When the employment status of the individuals who participated in the study was examined, 26·6 % of them reported working outside the home and 73·4 % of them reported working inside the home. It was found that there was a significant difference between the working status of the participants according to the total e-HDLQ (*t* = 3·546; *P* ˂ 0·001) and accessing sub-dimension (*t* = 4·154; *P* ˂ 0·001) scores. The total e-HDLQ scale scores of individuals working outside the home (X = 31·72) were higher than those of individuals working inside the home (X = 28·53) (*P* < 0·001). Among the participants, 66·6 % stated that their income was equal to their expenses, 15·9 % stated that their income was below their expenses and 17·5 % stated that their income was above their expenses. It was found that there was a significant difference between the total e-HDLQ score and income status (F = 7·116; *P* ˂ 0·001) (Table 1, see online supplementary material, Supplemental 1).

**Table 1.** Comparison of sociodemographic characteristics of pregnant women and scale scores

Characteristics	*n*	%	Total e-HDLQ		Total SNBS	
			Mean	sd	Mean	sd
Age groups						
18–32 years	229	79·0	29·35	6·73	108·25	17·41
33 years and over	61	21·0	27·91	5·27	112·68	15·35
* t*			−0·122		−1·810	
* P*			0·903		0·071	
Education level						
Primary education^a^	76	26·2	26·69	6·44	111·96	17·94
Secondary education^b^	113	39·0	29·26	6·88	107·69	15·62
Higher education^c^	101	34·8	31·52	6·61	108·77	17·86
F			11·354		1·471	
* P*			**˂ 0·001**		0·231	
Difference			**a ˂ b ˂ c**			
Spouse’s education level						
Primary education^a^	69	23·8	27·44	5·99	108·26	16·17
Secondary education^b^	117	40·3	28·76	7·14	108·55	17·15
Higher education^c^	104	35·9	31·34	6·78	110·50	17·64
F			7·692		0·492	
* P*			**0·001**		0·612	
Difference			**a ˂ b ˂ c**			
Working status outside the home						
Yes	77	26·6	31·72	6·51	108·28	19·02
No	213	73·4	28·53	6·87	109·51	16·35
* t*			3·546		−0·539	
* P*			**˂ 0·001**		0·590	
Income status						
Income below expenditure^a^	46	15·9	26·00	5·54	111·06	19·58
Income equal to expenditure^b^	193	66·6	30·19	6·97	108·58	16·30
Income over expenditure^c^	51	17·5	29·35	6·96	109·78	17·74
F			7·116		0·430	
* P*			**˂ 0·001**		0·651	
Difference			**a ˂ c ˂ b**			

e-HDLQ, e-healthy diet literacy scale; SNBS, Sustainable Nutrition Behavior Scale; *t*, *t* test for independent groups; F, ANOVA test, analysed at *P* ˂ 0·05 significance level. In the table, the letters a, b and c are used to indicate the difference between groups. *P* values indicated in bold are significant.

In the study, it was examined whether there was a statistical significance according to the scale scores of the obstetric characteristics of the participants. The mean gestational week is 32·22 (sd 6·71) (min = 12, max = 40). It was found that there was a statistically significant difference between the history of participants’ having a low-birth-weight baby and the total e-HDLQ (*t* = –2·882; *P* = 0·004) and appraising sub-dimension (*t* = –2·494; *P* = 0·013) scores. It was found that there was a significant difference between the number of pregnancies of the participants and the accessing sub-dimension score (F = 6·306; *P* = 0·002). The food preference sub-dimension score of overweight pregnant women (X = 22·64) was higher than that of obese pregnant women (X = 21·08) (Table 2, see online supplementary material, Supplemental 2).

**Table 2.** Comparison of obstetric characteristics of pregnant women and scale scores Table 2 long description.

Characteristics	*n*	%	Total e-HDLQ		Total SNBS	
			Mean	sd	Mean	sd
Gestational week						
12–28 weeks	58	20·0	29·55	6·37	109·06	18·61
29–40 weeks	232	80·0	29·33	7·05	109·21	16·71
*t*			0·212		−0·058	
* P*			0·832		0·954	
History of having a low-birth-weight baby						
Yes	49	16·9	26·81	6·85	107·81	21·49
No	241	83·1	29·90	6·82	109·46	16·06
*t*			−2·882		−0·509	
* P*			**0·004**		0·613	
Number of pregnancies						
1^a^	111	38·3	29·86	6·42	107·95	16·68
2^b^	85	29·3	29·40	7·51	109·08	17·22
3 and over ^c^	94	32·4	28·78	6·93	110·73	17·45
F			0·617		0·675	
* P*			0·540		0·510	
BMI during pregnancy						
Normal^a^	67	23·1	29·29	6·84	107·10	18·71
Overweight^b^	124	42·8	29·25	7·27	111·80	16·84
Obese^c^	99	34·1	29·59	6·56	107·31	15·89
F			0·074		2·581	
* P*			0·928		0·077	

e-HDLQ, e-healthy diet literacy scale; SNBS, Sustainable Nutrition Behavior Scale; *t*, *t* test for independent groups; F, ANOVA test, analysed at *P* ˂ 0·05 significance level. In the table, the letters a, b and c are used to indicate the difference between groups. *P* values indicated in bold are significant.

Among the participants, it was found that those who used the internet for 4 h or more (X = 6·83) had higher accessing sub-dimension scores than those who used the internet for less than 1 h (X = 5·75) (F = 3·767; *P* = 0·024). The total e-HDLQ (*t* = 2·813; *P* = 0·005), accessing sub-dimension score (*t* = 4·488; *P* = 0·001) and applying sub-dimension scores (*t* = 2·751; *P* = 0·006) were found to be statistically significant with the individuals’ knowledge of the concept of sustainable nutrition. While 22·8 % of the participants had received nutrition education before, the rest of the participants (77·2 %) had never received nutrition education before. The total e-HDLQ (*t* = 3·274; *P* = 0·001), accessing sub-dimension (*t* = 4·556; *P* ˂ 0·001) and applying sub-dimension (*t* = 2·734; *P* = 0·007) scores of the participants who had received nutrition education before were higher than those who had never received nutrition education. The total e-HDLQ (*t* = 3·794; *P* ˂ 0·001), accessing sub-dimension (*t* = 4·651; *P* ˂ 0·001) and applying sub-dimension (*t* = 3·186; *P* = 0·002) scores of the individuals with previous nutrition knowledge were higher than those with no nutrition knowledge. According to the study, when the participants were asked where they learned nutrition information, it was found that the most common source of nutrition information was from health professionals (41·7 %), followed by the internet (35·9 %) and finally from family, friends and environment (22·4 %) (Table 3, see online supplementary material, Supplemental 3).

**Table 3.** Evaluation of pregnant women’s characteristics of access to nutrition information according to scale scores

Characteristics	*n*	%	Total e-HDLQ		Total SNBS	
			Mean	sd	Mean	sd
Time spent on internet						
0–1 h^a^	61	21·0	28·44	6·69	110·91	17·27
2–3 h^b^	137	47·2	29·64	7·36	109·25	16·83
4 h and over^c^	92	31·7	29·60	6·35	107·93	17·37
F			0·708		0·560	
* P*			0·493		0·572	
Knowledge of sustainable nutrition concept						
Yes	64	22·1	31·50	7·14	108·92	16·04
No	226	77·9	28·77	6·74	109·26	17·39
*t*			2·813		−0·140	
* P*			**0·005**		0·889	
Nutrition education status						
Yes	66	22·8	31·78	6·54	109·33	16·26
No	224	77·2	28·66	6·87	109·14	17·34
*t*			3·274		0·080	
* P*			**0·001**		0·937	
Knowledge of nutrition						
Yes	137	47·2	30·97	6·90	108·94	17·51
No	153	52·8	27·95	6·62	109·40	16·72
*t*			3·794		−0·230	
* P*			**˂ 0·001**		0·818	
Source of nutrition information						
Family and friends^a^	65	22·4	28·15	6·83	107·92	16·08
Health professionals^b^	121	41·7	30·27	7·08	109·83	17·13
Internet^c^	104	35·9	29·10	6·68	109·22	17·72
F			2·129		0·264	
* P*			0·121		0·768	

e-HDLQ, e-healthy diet literacy scale; SNBS, Sustainable Nutrition Behavior Scale; *t*, *t* test for independent groups; F, ANOVA test, analysed at *P* ˂ 0·05 significance level. In the table, the letters a, b and c are used to indicate the difference between groups. *P* values indicated in bold are significant.

It was determined that there was a statistical relationship between the total e-HDLQ score of the participants and the SNBS food preference (*r* = 0·119) and food purchase (*r* = 0·117) sub-dimensions (*P* ˂ 0·05). A statistical relationship was found between the e-HDLQ accessing sub-dimension and the SNBS food purchase sub-dimension (*r* = 0·182, *P* ˂ 0·01). It was determined that there was a statistical relationship between the total e-HDLQ appraising sub-dimension and total SNBS score (*r* = 0·125), food preference (*r* = 0·134) sub-dimension and food waste reduction (*r* = 0·119) sub-dimension (*P* ˂ 0·05) (Table 4).

**Table 4.** Examination of the relationship between the scale scores of the participants

	Scales		e-HDLQ sub-dimensions			
		Total e-HDLQ	Accessing	Understanding	Appraising	Applying
	Total SNBS	0·079	0·094	−0·025	**0·125** ^*^	0·085
SNBS sub-dimensions	Food preference	0·119*	0·095	0·025	**0·134** ^*^	0·095
	Food waste reduction	0·049	0·037	−0·031	**0·119** ^*^	0·075
	Seasonal and local food consumption	−0·012	0·008	−0·069	0·102	0·001
	Food purchase	**0·117** ^*^	**0·182** ^**^	0·005	0·056	0·114

e-HDLQ, e-healthy diet literacy scale; SNBS, Sustainable Nutrition Behavior Scale. *P* values indicated in bold are significant. *˂ 0·05, **˂ 0·01.

Two multiple linear regression models were conducted using the Backward method to examine the independent predictors of e-HDLQ and SNBS, controlling for sociodemographic and obstetric variables. Model 1 (e-HDLQ**)** was statistically significant and explained 17·3 % of the variance in total scores (R^2^ = 0·173, F(7, 282) = 8·400, *P* < 0·001; Durbin–Watson = 1·466). Significant positive predictors included age, education level, history of having a low-birth-weight baby and the food preference and food purchase sub-dimensions. Conversely, higher knowledge of nutrition and the seasonal and local food consumption sub-dimension were associated with lower e-HDLQ scores. Model 2 (SNBS) was also significant, although it explained a smaller proportion of variance (R^2^ = 0·034, F(3, 286) = 3·404, *P* = 0·018; Durbin–Watson = 1·505). The appraising sub-dimension was a positive predictor of SNBS, while higher education level was negatively associated with sustainable nutrition behaviour. The accessing sub-dimension was not statistically significant (Table 5).

**Table 5.** Multiple linear regression analysis predicting total e-HDLQ and total SNBS

Variable	B	Std. error	*β*	*t*	*P*	95 % CI (Lower, Upper)	Tolerance	VIF
Predictors of total e-HDLQ								
Age	0·172	0·075	0·131	2·289	**0·023**	0·024, 0·320	0·893	1·120
Education level	1·931	0·512	0·217	3·772	**<0·001**	0·924, 2·939	0·884	1·131
History of having a low-birth-weight baby	3·202	1·046	0·174	3·060	**0·002**	1·143, 5·262	0·909	1·100
Knowledge of nutrition	−2·146	0·781	−0·155	−2·750	**0·006**	−3·683, –0·610	0·921	1·086
Food preference	0·289	0·105	0·182	2·758	**0·006**	0·083, 0·495	0·672	1·487
Seasonal and local food consumption	−0·266	0·095	−0·209	−2·792	**0·006**	−0·454, –0·079	0·523	1·912
Food purchase	0·205	0·093	0·152	2·195	**0·029**	0·021, 0·388	0·616	1·624
Model 1: R = 0·415; R^2^ = 0·173; F(7, 282) = 8·400, *P* < 0·001; Durbin–Watson = 1·466								
Predictors of total SNBS								
Education level	−2·681	1·350	−0·122	−1·987	**0·048**	−5·338, –0·025	0·893	1·119
Accessing	0·732	0·413	0·109	1·772	0·078	−0·081, 1·546	0·900	1·112
Appraising	1·020	0·469	0·130	2·176	**0·030**	0·097, 1·942	0·951	1·052
Model 2: R = 0·186; R^2^ = 0·034; F(3, 286) = 3·404, *P* = 0·018; Durbin–Watson = 1·505								

Backward Model in Multiple Linear Regression; e-HDLQ, e-healthy diet literacy scale; SNBS, Sustainable Nutrition Behavior Scale; VIF, Variance Inflation Factor. *P* values indicated in bold are significant.

## Discussion

### Discussion of the main results

In this study, e-healthy diet literacy levels of pregnant women were moderate (29·37 (sd 6·91)). This is consistent with the scoring range of the tool and with the results of similar studies conducted in different populations. In a study conducted on adult individuals, the e-HDLQ score of women was (29·2 (sd 6·4)), which is similar to this study^(25)^.

In the study, the total scores, accessing, appraising and applying sub-dimensions of e-HDLQ of higher education graduates were found to be higher than those of primary education graduates. Women with higher levels of education may have easier access to current health-related research and accurate information, which may facilitate the acquisition of evidence-based information on nutrition. As in this study, in other studies in the literature, the level of nutrition knowledge increased as the level of education increased^(26–28)^. However, although the associations were statistically significant, the effect sizes were minimal. This suggests that these relationships should be interpreted with caution, rather than as evidence of significant behavioural change.

In our study, although pregnant women working outside the home were in the minority, the e-HDLQ scores (31·72 (sd 6·51)) and accessing (7·51 (sd 2·47)) sub-dimensions were higher. This situation may partly reflect higher educational attainment among women working outside the home, as education is strongly linked to both digital and nutrition literacy^(29,30)^. Therefore, the observed difference may be influenced by the interplay between education, working status and access to trustworthy information rather than working status alone. Women working outside the home (26·6 %) may have more regular eating habits than women working inside the home. In this case, it may reveal situations such as obtaining healthy nutrition information and access to the right source. In this study, as the income level increased, the score of e-HDLQ also increased. In one study, those with low nutritional literacy were two times more likely to have low annual household income^(31)^. In another study, in contrast to our findings, although most of the participants were low-income individuals, their access to information and literacy levels were high^(28)^. This suggests that income alone does not determine digital or nutrition literacy and that other factors such as health literacy, digital literacy, cultural norms, and nutrition education may also play an important role.

In this study, the accessing sub-dimension score of women with first pregnancy was found to be higher. According to the study of Papežová *et al.* (2023), the e-healthy diet literacy of women with first pregnancy was found to be higher^(32)^. The food preference score of overweight pregnant women was higher than that of obese pregnant women. Obese pregnant women’s special diets for metabolic diseases may have influenced their lower scores compared to the flexible choices of overweight women. In this study, the characteristics of pregnant women’s access to nutrition information and scale scores were compared. As the duration of internet use increased, the accessing sub-dimension scores increased. When the studies were reviewed, most of the pregnant women searched on the internet to meet their information needs^(33–35)^. There is also evidence showing that women actively use the internet for sustainable nutrition and healthy lifestyle information^(34–36)^.

Pregnant women who knew the concept of sustainable nutrition before had higher total e-HDLQ, accessing and applying sub-dimension scores than those who did not. Pregnant women who received nutrition education or who had previously received nutrition information had higher total e-HDLQ scores, accessing and applying sub-dimension scores than those who did not receive nutrition education. The primary source of nutrition information was health professionals (41·7 %). The applying sub-dimension scores and the source of nutrition information were also found to be statistically significant. Looking at the studies, it was observed that pregnant women who received nutrition information and nutrition education had high levels of healthy nutrition literacy^(32,37,38)^. This may be because nutrition education interventions increase pregnant women’s awareness of nutrient intake. Our study findings showed that participation in pregnancy-related educational activities was low (22·8 %). In the literature, it is stated that participation in childbirth education courses has decreased, and these courses have positive effects on pregnancy outcomes^(39–41)^. Therefore, it is recommended that such courses reach a wider audience by integrating the use of the internet. Studies have shown that the main source of nutrition information is health professionals^(42,43)^, while online resources are increasingly used as complementary information channels^(43–45)^.

According to our study, as the age of the participants increased, their food purchase sub-dimension scores increased. As age increases, awareness of environmental problems and climate change increases, which may encourage sustainable consumption habits. Sustainable consumption can be seen as a means of presenting a positive image and counteracting negative age-related perceptions^(46)^.

In our study, a relationship was found between total e-HDLQ and food preference sub-dimension. Given the weak correlation coefficients in this section, these associations indicate slight tendencies rather than strong behavioural changes. Clear food labels with nutritional values and origins help consumers make informed decisions, enhance food literacy and provide accurate nutrition information. Although most people try to examine the nutrition information on labels while shopping, they may have difficulty in understanding all of this information^(47,48)^. A relationship was found between the food purchase sub-dimension and total e-HDLQ. Pregnant women can make healthy and appropriate choices by better understanding the content of their foods thanks to digital resources. A relationship was found between the food purchase sub-dimension and the accessing sub-dimension. Easy access to healthy foods enables pregnant women to eat regularly and make healthier purchasing choices. The analysis indicated that the appraising sub-dimension plays a central role within the scale structure. This component was positively linked not only with the overall SNBS score but also with specific behavioural domains such as food preference and food waste reduction. Taken together, these associations suggest that individuals with stronger appraisal skills tend to exhibit more favourable sustainable nutrition practices.

Importantly, multiple linear regression analyses indicated that age, education level, history of having a low-birth-weight baby and the food preference and food purchase sub-dimensions were significant positive predictors of e-HDLQ, whereas knowledge of nutrition and the seasonal and local food consumption sub-dimension were negatively associated with e-HDLQ scores. Similarly, another study found a positive correlation between age, educational level and nutritional literacy^(29)^.

For sustainable nutrition behaviour, the appraising sub-dimension was a positive predictor, and higher education level was negatively associated, whereas the accessing sub-dimension was not significant. However, contrary to the present findings regarding education and sustainable nutrition behaviours, previous research has reported a positive association between higher education and sustainable nutrition behaviours^(49)^.

A recent study found that participants with formal nutrition education exhibited more favourable sustainable nutrition behaviours^(50)^.

Finally, future research should explore opportunities to promote environmentally sustainable foods during pregnancy, considering their acceptability and popularity. Healthcare professionals, including nutritionists and dietitians, should emphasise the benefits of sustainable foods and provide practical guidance, such as recipes, to incorporate them into diets.

### Limitations

The data collection area was limited to home visits to pregnant women residing in Selçuklu/Konya District Governorate. Data collection through home visits had limitations such as time and access constraints, confidentiality concerns and logistical difficulties (such as difficult access, traffic and road conditions). However, it should be noted that this district is a densely populated urban centre with generally easy access to healthcare services and no internet access problems. These characteristics may be associated with higher e-health diet literacy and more sustainable nutrition behaviour scores, compared with populations living in rural or less developed areas. Because the study used convenience sampling from a single district, the findings may not be generalisable to all pregnant women in Türkiye, limiting external validity.

As a cross-sectional study, causality cannot be inferred, and the observed associations (particularly those with minimal effect sizes) should be interpreted cautiously. Potential confounding factors, including socio-economic status, place of residence, general digital literacy and other social determinants, were not controlled for in the analyses. These variables may have contributed to the variability observed in the associations, and future research should consider incorporating them to better clarify their role in e-healthy diet literacy and sustainable nutrition behaviours. Although multiple linear regression was conducted to examine independent predictors, residual confounding may still exist due to unmeasured variables.

Another important limitation is that all data were based on self-reported questionnaires. Self-reported measures are subject to recall bias and social desirability bias, which may have led participants to overestimate healthy behaviours or underestimate unhealthy ones.

The relatively low internal consistency of the e-HDLQ in this study limits the interpretability of results and suggests caution in generalising results. Therefore, results related to the e-HDLQ should be interpreted as indicative rather than definitive, and future research should consider revised versions or alternative instruments with higher reliability.

### Implication for practice

Based on the findings of this study, which indicate statistically significant but weak associations, digital tools such as mobile applications, tele-nutrition counselling or online educational platforms may offer potential avenues to support pregnant women in accessing reliable nutrition information. Although the current results do not provide strong evidence for recommending these interventions as effective strategies, future research could explore whether digital approaches contribute to improvements in e-healthy diet literacy and sustainable nutrition behaviours. Healthcare professionals might consider guiding pregnant women towards credible digital resources and strengthening patient-centred communication, particularly for those with lower digital literacy levels. Such practices could help address gaps in accessing accurate nutrition information; however, their effectiveness should be empirically tested in future intervention studies. Overall, e-healthy diet literacy may play a modest role in shaping sustainable eating habits during pregnancy, but the weak effect sizes observed suggest that multiple social, environmental and individual factors likely influence these behaviours. Future research should examine how these contextual factors interact with digital literacy to shape nutrition-related decision-making.

### Conclusions

In conclusion, e-healthy diet literacy during pregnancy may play a modest role in supporting more conscious and sustainable eating habits. However, given the weak associations identified in this study, these findings should not be interpreted as evidence of a strong or causal relationship. Rather, they suggest that multiple social, environmental and individual factors likely influence pregnant women’s nutrition-related behaviours. Future studies are warranted to further explore the potential contribution of digital diet literacy within broader contextual determinants.

As the primary results of the study, a positive relationship was found between e-healthy diet literacy and sustainable nutrition behaviours. The results are statistically significant, but the relationship is quite weak. This suggests that other things, not just how well pregnant women understand digital health information, may affect how they behave in terms of nutrition. In future studies, other social and environmental factors should be considered.

The regression results showed that e-HDLQ was positively predicted by age, education level, history of having a low-birth-weight baby and the sub-dimensions of food preference and food purchase. For SNBS, the appraising sub-dimension was a positive predictor, while higher education level was negatively associated. These findings highlight the differential contributions of specific sociodemographic and behavioural factors in shaping both digital diet literacy and sustainable nutrition practices among pregnant women.

Secondary results showed that participants aged 33 years and over had a statistically higher food purchase sub-dimension score than participants aged 18–32 years. The total and sub-dimension scores of e-HDLQ of pregnant women with higher education and their spouses were found to be higher than the groups with lower education levels. Pregnant women who worked outside the home, whose income was equal to their expenditures and who lived in metropolitan areas had higher e-HDLQ scores, while those who lived in towns had higher SNBS and food purchase scores than those who lived in metropolitan areas. Those who did not have a low-birth-weight baby before and those who received nutrition education had higher e-HDLQ scores. In addition, those who knew the concept of sustainable nutrition, those who were overweight and those who used healthcare professionals as a source of information had higher scores. It was determined that access scores increased as the duration of internet use increased.

## Supporting information

10.1017/S1368980026102870.sm001 Demir and Erkal Aksoy supplementary material Demir and Erkal Aksoy supplementary material

## Data Availability

The data underlying this article will be shared on reasonable request to the corresponding author. The data that support the findings of this study are available from the corresponding author (SRG) upon reasonable request.

## Supplementary material

For supplementary material accompanying this paper visit https://doi.org/10.1017/S1368980026102870

## Statistics information

The author(s) affirm that the methods used in the data analyses are suitably applied to their data within their study design and context, and the statistical findings have been implemented and interpreted correctly. The author(s) agrees to take responsibility for ensuring that the choice of statistical approach is appropriate and is conducted and interpreted correctly as a condition to submit to the Journal.

## Table 2. Long description

The table compares obstetric characteristics of pregnant women and their scale scores. It has 12 rows and 7 columns. The columns are labeled Characteristics, n, percent, Total e-HDLQ Mean, Total e-HDLQ SD, Total SNBS Mean, and Total SNBS SD. The rows are labeled with different obstetric characteristics such as Gestational week, History of having a low-birth-weight baby, Number of pregnancies, and BMI during pregnancy. Each characteristic is further divided into subcategories with corresponding values for n, percent, Total e-HDLQ Mean, Total e-HDLQ SD, Total SNBS Mean, and Total SNBS SD. Notable trends include statistical significance in the history of having a low-birth-weight baby and the number of pregnancies.

Navigate back to Table 2..
