# Supplementary material for: The effect of e-healthy diet literacy on sustainable nutrition behaviours in pregnant women
Source: Public Health Nutr. 2026 Jun 16;29(1):e149. doi: 10.1017/S1368980026102870 (PMC13434611; doi:10.1017/S1368980026102870)
Supplement: Supplementary file 1 [file S1368980026102870sup001.docx]

**Supplementary 1. Comparison of sociodemographic characteristics of pregnant women and sub-dimensions scores**

|  | **e-HDLQ Sub-dimensions** | | | | | | | | **SNBS Sub-dimensions** | | | | | | | |
| --- | --- | --- | --- | --- | --- | --- | --- | --- | --- | --- | --- | --- | --- | --- | --- | --- |
| **Characteristics** | **Accessing** | | **Understanding** | | **Appraising** | | **Applying** | | **Food Preference** | | **Food Waste Reduction** | | **Seasonal and Local Food Consumption** | | **Food Purchase** | |
|  | **Mean** | **SD** | **Mean** | **SD** | **Mean** | **SD** | **Mean** | **SD** | **Mean** | **SD** | **Mean** | **SD** | **Mean** | **SD** | **Mean** | **SD** |
| **Age Groups** | | | | | | | | | | | | | | | | |
| 18-32 years | 6.52 | 2.54 | 10.37 | 4.64 | 7.14 | 2.18 | 5.30 | 1.74 | 21.70 | 4.54 | 34.65 | 5.98 | 30.28 | 5.49 | 21.61 | 5.31 |
| 33 years and over | 6.49 | 2.48 | 10.55 | 5.00 | 6.95 | 2.10 | 5.47 | 1.94 | 22.13 | 3.60 | 35.27 | 5.93 | 31.75 | 5.04 | 23.52 | 3.99 |
| **t** | 0.100 | | -0.267 | | 0.631 | | -0.675 | | -0.673 | | -0.730 | | -1.888 | | -3.086 | |
| **p** | 0.920 | | 0.789 | | 0.528 | | 0.500 | | 0.501 | | 0.466 | | 0.060 | | **0.003** | |
| **Education Level** | | | | | | | | | | | | | | | | |
| Primary education^a^ | 5.64 | 2.59 | 9.73 | 4.68 | 6.51 | 2.15 | 4.80 | 1.97 | 22.21 | 4.85 | 35.65 | 6.018 | 31.43 | 5.61 | 22.65 | 5.62 |
| Secondary education^b^ | 6.21 | 2.29 | 10.76 | 4.78 | 7.09 | 2.05 | 5.19 | 1.59 | 21.40 | 3.91 | 34.64 | 5.57 | 30.07 | 5.10 | 21.56 | 4.89 |
| Higher education^c^ | 7.52 | 2.41 | 10.53 | 4.65 | 7.56 | 2.21 | 5.90 | 1.71 | 21.92 | 4.45 | 34.27 | 6.33 | 30.54 | 5.60 | 22.02 | 4.96 |
| **F** | 14.600 | | 1.124 | | 5.236 | | 9.269 | | 0.832 | | 1.211 | | 1.444 | | 1.034 | |
| **p** | **<0.001** | | 0.326 | | **0.006** | | **<0.001** | | 0.436 | | 0.300 | | 0.238 | | 0.357 | |
| **Difference** | **a,b˂c** | |  | | **a<c** | | **a,b˂c** | |  | |  | |  | |  | |
| **Spouse’s Education** | | | | | | | | | | | | | | | | |
| Primary education^a^ | 5.75 | 2.43 | 10.08 | 4.37 | 6.81 | 2.14 | 4.79 | 1.64 | 21.44 | 4.26 | 34.62 | 5.74 | 30.42 | 4.97 | 21.76 | 5.53 |
| Secondary education^b^ | 6.35 | 2.42 | 10.42 | 4.92 | 6.79 | 2.21 | 5.19 | 1.90 | 21.95 | 4.26 | 34.70 | 5.96 | 30.17 | 5.50 | 21.70 | 4.96 |
| Higher education^c^ | 7.22 | 2.54 | 10.61 | 4.72 | 7.65 | 2.04 | 5.85 | 1.62 | 21.84 | 4.55 | 34.97 | 6.16 | 31.17 | 5.62 | 22.51 | 5.02 |
| **F** | 7.768 | | 0.260 | | 5.302 | | 8.272 | | 0.303 | | 0.085 | | 0.968 | | 0.792 | |
| **p** | **0.001** | | 0.771 | | **0.005** | | **<0.001** | | 0.739 | | 0.919 | | 0.381 | | 0.454 | |
| **Difference** | **a,b˂c** | |  | | **a,b˂c** | | **a,b˂c** | |  | |  | |  | |  | |
| **Working Outside Home** | | | | | | | | | | | | | | | | |
| Yes | 7.51 | 2.47 | 11.11 | 4.61 | 7.41 | 2.17 | 5.67 | 1.75 | 21.90 | 4.33 | 34.00 | 6.60 | 30.28 | 5.56 | 22.09 | 5.30 |
| No | 6.15 | 2.45 | 10.15 | 4.73 | 6.99 | 2.16 | 5.21 | 1.78 | 21.75 | 4.38 | 35.06 | 5.71 | 30.70 | 5.38 | 21.98 | 5.06 |
| **t** | 4.154 | | 1.531 | | 1.459 | | 1.940 | | 0.264 | | -1.345 | | -0.579 | | 0.154 | |
| **p** | **<0.001** | | 0.127 | | 0.146 | | 0.053 | | 0.792 | | 0.180 | | 0.563 | | 0.878 | |
| **Income Status** | | | | | | | | | | | | | | | | |
| Income below expenditure^a^ | 5.97 | 2.47 | 8.52 | 4.09 | 6.63 | 2.59 | 4.86 | 1.79 | 22.34 | 4.59 | 35.30 | 6.93 | 31.15 | 5.88 | 22.26 | 5.82 |
| Income equal to expenditure^b^ | 6.52 | 2.49 | 10.94 | 4.86 | 7.20 | 2.00 | 5.51 | 1.76 | 21.70 | 4.20 | 34.67 | 5.59 | 30.35 | 5.39 | 21.84 | 4.91 |
| Income over expenditure^c^ | 6.98 | 2.66 | 10.11 | 4.25 | 7.15 | 2.33 | 5.09 | 1.80 | 21.64 | 4.78 | 34.72 | 6.48 | 31.00 | 5.15 | 22.41 | 5.29 |
| **F** | 1.911 | | 5.167 | | 1.331 | | 3.000 | | 0.438 | | 0.209 | | 0.576 | | 0.305 | |
| **p** | 0.150 | | **0.006** | | 0.266 | | 0.051 | | 0.646 | | 0.811 | | 0.563 | | 0.737 | |
| **Difference** |  | | **a<b** | |  | |  | |  | |  | |  | |  | |

*Note: t: t-test for independent groups. F: ANOVA test (analysis of variance), analyzed at p˂0.05 significance level. In the table, the letters a,b,c are used to indicate the difference between groups. p values indicated in bold are significant.*

**Supplementary 2. Comparison of obstetric characteristics of pregnant women and sub-dimensions scores**

|  | **e-HDLQ Sub-dimensions** | | | | | | | | | **SNBS Sub-dimensions** | | | | | | | |
| --- | --- | --- | --- | --- | --- | --- | --- | --- | --- | --- | --- | --- | --- | --- | --- | --- | --- |
| **Characteristics** | **Accessing** | | **Understanding** | | **Appraising** | | **Applying** | | **Food Preference** | | | **Food Waste Reduction** | | **Seasonal and Local Food Consumption** | | **Food Purchase** | |
|  | **Mean** | **SD** | **Mean** | **SD** | **Mean** | **SD** | **Mean** | **SD** | **Mean** | | **SD** | **Mean** | **SD** | **Mean** | **SD** | **Mean** | **SD** |
| **Gestational Week** | | | | | | | | | | | | | | | | | |
| 12 to 28 weeks | 6.72 | 2.72 | 10.27 | 4.43 | 6.91 | 2.05 | 5.63 | 1.74 | 21.70 | | 4.26 | 35.31 | 6.23 | 29.74 | 6.04 | 22.31 | 4.93 |
| 29 to 40 weeks | 6.46 | 2.48 | 10.44 | 4.78 | 7.15 | 2.20 | 5.26 | 1.79 | 21.81 | | 4.39 | 34.65 | 5.90 | 30.80 | 5.25 | 21.93 | 5.17 |
| **t** | 0.684 | | -0.249 | | -0.757 | | 1.430 | | -0.175 | | | 0.752 | | -1.338 | | 0.493 | |
| **p** | 0.495 | | 0.804 | | 0.450 | | 0.154 | | 0.861 | | | 0.453 | | 0 .182 | | 0 .623 | |
| **History of Having a Low-Birth-Weight Baby** | | | | | | | | | | | | | | | | | |
| Yes | 5.95 | 2.76 | 9.30 | 4.42 | 6.40 | 2.20 | 5.14 | 1.96 | 21.95 | | 4.46 | 33.83 | 7.32 | 30.00 | 6.71 | 22.02 | 5.53 |
| No | 6.63 | 2.47 | 10.63 | 4.74 | 7.24 | 2.13 | 5.37 | 1.75 | 21.76 | | 4.35 | 34.97 | 5.65 | 30.71 | 5.13 | 22.01 | 5.04 |
| **t** | -1.710 | | -1.811 | | -2.494 | | -0.837 | | 0.286 | | | -1.218 | | -0.704 | | 0.010 | |
| **p** | 0.088 | | 0.071 | | **0.013** | | 0.403 | | 0.775 | | | 0.224 | | 0.484 | | 0.992 | |
| **Number of Pregnancies** | | | | | | | | | | | | | | | | | |
| 1^a^ | 6.98 | 2.43 | 10.05 | 4.63 | 7.36 | 2.18 | 5.45 | 1.61 | 21.75 | | 4.59 | 34.30 | 5.83 | 30.31 | 5.27 | 21.57 | 4.95 |
| 2^b^ | 6.72 | 2.65 | 10.40 | 4.58 | 7.18 | 2.13 | 5.08 | 1.84 | 21.98 | | 4.52 | 34.65 | 5.72 | 30.63 | 5.47 | 21.80 | 5.22 |
| 3 and over ^c^ | 5.78 | 2.38 | 10.85 | 4.92 | 6.72 | 2.15 | 5.42 | 1.92 | 21.67 | | 3.96 | 35.45 | 6.33 | 30.88 | 5.60 | 22.72 | 5.20 |
| **F** | 6.306 | | 0.727 | | 2.360 | | 1.238 | | 0.125 | | | 0.972 | | 0.281 | | 1.385 | |
| **p** | **0.002** | | 0.484 | | 0.096 | | 0.291 | | 0.882 | | | 0.379 | | 0.756 | | 0.252 | |
| **Difference** | **a,b>c** | |  | |  | |  | |  | | |  | |  | |  | |
| **BMI during Pregnancy** | | | | | | | | | | | | | | | | | |
| Normal^a^ | 6.65 | 2.72 | 9.79 | 4.68 | 7.41 | 1.70 | 5.43 | 1.93 | 21.28 | | 4.61 | 34.29 | 6.15 | 30.07 | 5.51 | 21.44 | 5.44 |
| Overweight^b^ | 6.59 | 2.37 | 10.12 | 4.48 | 7.14 | 2.23 | 5.37 | 1.75 | 22.64 | | 4.17 | 35.37 | 5.67 | 31.14 | 5.60 | 22.64 | 4.93 |
| Obese^c^ | 6.33 | 2.60 | 11.19 | 4.94 | 6.84 | 2.35 | 5.22 | 1.74 | 21.08 | | 4.27 | 34.37 | 6.19 | 30.25 | 5.11 | 21.60 | 5.09 |
| **F** | 0.423 | | 2.177 | | 1.413 | | 0.333 | | 4.234 | | | 1.056 | | 1.143 | | 1.674 | |
| **p** | 0.656 | | 0.115 | | 0.245 | | 0.717 | | **0.015** | | | 0.349 | | 0.320 | | 0.189 | |
| **Difference** |  | |  | |  | |  | | **c˂b** | | |  | |  | |  | |

*Note: t: t-test for independent groups. F: ANOVA test (analysis of variance), analyzed at p˂0.05 significance level. In the table, the letters a,b,c are used to indicate the difference between groups. p values indicated in bold are significant.*

**Supplementary 3. Evaluation of Pregnant Women's Characteristics of Access to Nutrition Information According to Sub-dimensions Scores**

|  | **e-HDLQ Sub-dimensions** | | | | | | | | **SNBS Sub-dimensions** | | | | | | | |
| --- | --- | --- | --- | --- | --- | --- | --- | --- | --- | --- | --- | --- | --- | --- | --- | --- |
| **Characteristics** | **Accessing** | | **Understanding** | | **Appraising** | | **Applying** | | **Food Preference** | | **Food Waste Reduction** | | **Seasonal and Local Food Consumption** | | **Food Purchase** | |
|  | **Mean** | **SD** | **Mean** | **SD** | **Mean** | **SD** | **Mean** | **SD** | **Mean** | **SD** | **Mean** | **SD** | **Mean** | **SD** | **Mean** | **SD** |
| **Time Spent on Internet** | | | | | | | | | | | | | | | | |
| 0-1 hour^a^ | 5.75 | 2.27 | 10.75 | 5.14 | 6.67 | 2.16 | 7.10 | 2.17 | 22.47 | 3.85 | 35.24 | 6.07 | 30.88 | 5.40 | 22.31 | 5.67 |
| 2-3 hours^b^ | 6.64 | 2.58 | 10.27 | 4.54 | 7.27 | 2.13 | 5.43 | 1.92 | 21.86 | 4.27 | 34.59 | 6.10 | 30.51 | 5.64 | 22.27 | 4.84 |
| 4 and over^c^ | 6.83 | 2.53 | 10.39 | 4.70 | 7.14 | 2.21 | 5.23 | 1.60 | 21.23 | 4.75 | 34.75 | 5.72 | 30.52 | 5.16 | 21.42 | 5.14 |
| **F** | 3.767 | | 0.216 | | 1.665 | | 0.407 | | 1.513 | | 0.249 | | 0.111 | | 0.895 | |
| **p** | **0.024** | | 0.806 | | 0.191 | | 0.666 | | 0.222 | | 0.780 | | 0.895 | | 0.410 | |
| **Difference** | **a˂c** | |  | |  | |  | |  | |  | |  | |  | |
| **Knowledge of Sustainable Nutrition Concept** | | | | | | | | | | | | | | | | |
| Yes | 7.73 | 2.48 | 10.37 | 4.43 | 7.51 | 1.91 | 5.87 | 1.62 | 21.96 | 4.19 | 34.28 | 5.77 | 30.14 | 5.45 | 22.53 | 4.16 |
| No | 6.17 | 2.44 | 10.42 | 4.79 | 6.99 | 2.22 | 5.18 | 1.80 | 21.74 | 4.41 | 34.92 | 6.02 | 30.72 | 5.42 | 21.86 | 5.35 |
| **t** | 4.488 | | -0.074 | | 1.712 | | 2.751 | | 0.357 | | -0.761 | | -0.755 | | 0.916 | |
| **p** | **0.001** | | 0.941 | | 0.088 | | **0.006** | | 0.721 | | 0.447 | | 0.451 | | 0.361 | |
| **Nutrition Education Status** | | | | | | | | | | | | | | | | |
| Yes | 7.72 | 2.38 | 10.72 | 4.71 | 7.53 | 2.00 | 5.80 | 1.48 | 21.83 | 4.42 | 34.45 | 5.95 | 30.43 | 4.52 | 22.60 | 4.74 |
| No | 6.16 | 2.46 | 10.32 | 4.72 | 6.98 | 2.20 | 5.20 | 1.85 | 21.78 | 4.35 | 34.87 | 5.98 | 30.63 | 5.67 | 21.83 | 5.22 |
| **t** | 4.556 | | 0.614 | | 1.810 | | 2.734 | | 0.078 | | -0.508 | | -0.261 | | 1.070 | |
| **p** | **˂0.001** | | 0.540 | | 0.071 | | **0 .007** | | 0 .938 | | 0.612 | | 0.794 | | 0.286 | |
| **Knowledge of Nutrition** | | | | | | | | | | | | | | | | |
| Yes | 7.22 | 2.54 | 10.89 | 4.81 | 7.16 | 2.22 | 5.68 | 1.73 | 21.56 | 4.27 | 34.84 | 6.22 | 30.44 | 5.44 | 22.08 | 5.15 |
| No | 5.88 | 2.34 | 9.98 | 4.59 | 7.05 | 2.12 | 5.02 | 1.78 | 22.00 | 4.44 | 34.72 | 5.74 | 30.72 | 5.43 | 21.95 | 5.10 |
| **t** | 4.651 | | 1.634 | | 0.452 | | 3.186 | | -0.839 | | 0.172 | | -0.438 | | 0.209 | |
| **p** | **˂0.001** | | 0.103 | | 0 .652 | | **0.002** | | 0.402 | | 0.863 | | 0.661 | | 0.835 | |
| **Source of Nutrition Information** | | | | | | | | | | | | | | | | |
| Family, friends^a^ | 6.52 | 2.84 | 10.21 | 4.63 | 6.84 | 2.23 | 4.56 | 1.69 | 21.06 | 4.16 | 33.89 | 6.11 | 30.89 | 4.90 | 22.07 | 4.62 |
| Health professionals^b^ | 6.66 | 2.41 | 10.57 | 4.84 | 7.27 | 2.17 | 5.76 | 1.64 | 21.86 | 4.03 | 35.11 | 5.89 | 30.37 | 5.37 | 22.47 | 5.34 |
| Internet^c^ | 6.35 | 2.47 | 10.34 | 4.65 | 7.07 | 2.12 | 5.32 | 1.86 | 22.17 | 4.80 | 34.95 | 5.96 | 30.66 | 5.81 | 21.43 | 5.13 |
| **F** | 0.406 | | 0.141 | | 0.831 | | 9.957 | | 1.329 | | 0.953 | | 0.207 | | 1.176 | |
| **p** | 0.667 | | 0.868 | | 0.437 | | **0.001** | | 0.266 | | 0.387 | | 0.813 | | 0.310 | |
| **Difference** |  | |  | |  | | **a˂c˂b** | |  | |  | |  | |  | |

*Note: t: t-test for independent groups. F: ANOVA test (analysis of variance), analyzed at p˂0.05 significance level. In the table, the letters a,b,c are used to indicate the difference between groups. p values indicated in bold are significant.*
